# Patent Foramen Ovale Closure for Stroke Prevention and Other Disorders

**DOI:** 10.1161/JAHA.117.007146

**Published:** 2018-06-17

**Authors:** Fareed Moses S. Collado, Marie‐France Poulin, Joshua J. Murphy, Hani Jneid, Clifford J. Kavinsky

**Affiliations:** ^1^ Division of Cardiology Department of Medicine Rush University Medical Center Chicago IL; ^2^ Division of Cardiology Department of Medicine Baylor College of Medicine Houston TX

**Keywords:** migraine, patent foramen ovale, patent foramen ovale closure, shunt, stroke, Catheter-Based Coronary and Valvular Interventions, Cerebrovascular Disease/Stroke, Congenital Heart Disease, Echocardiography

Stroke is the fifth‐most common cause of death and the leading cause of preventable adult disability in the United States.^1^ In 2013, there were 6.5 million stroke‐related deaths worldwide accounting for 11.8% of total deaths.^2^ Strokes cost $34 billion each year in the United States.^1^ This estimate includes the cost of health care, medications, and missed workdays.

Cryptogenic stroke is defined as brain infarction that is not attributed to definite large‐vessel atherosclerosis, small‐artery disease, or embolism despite extensive vascular, serological, and cardiac evaluation.^3^ Approximately one‐third of all ischemic strokes are considered cryptogenic.^4^ The causal relationship between patent foramen ovale (PFO) and cryptogenic stroke has historically been controversial. Approximately 25% of the adult population has a PFO, and the condition by itself has not been shown to increase the risk of ischemic stroke.^5^, ^6^ Yet, the prevalence of PFO is significantly higher in patients with cryptogenic stroke; up to 40% of ischemic strokes without an identifiable cause have a PFO.^7^ This suggests that paradoxical embolism through a PFO may be implicated in a proportion of cryptogenic strokes.

The association of PFO with stroke was first described in 1877 by Julius Friedrich Cohnheim, a German pathologist and protégé of Virchow. He performed a necropsy on a 35‐year‐old woman who had a fatal stroke and found a long thrombus in the lower extremity and as well as a “very large” foramen ovale, through which he could pass 3 fingers with ease. Cohnheim then hypothesized that a torn‐off piece of thrombus arising from the lower extremity traveled to the right atrium into the left atrium and to the frontal lobe.^8^

## PFO Definition and Diagnosis

**Figure 1:**
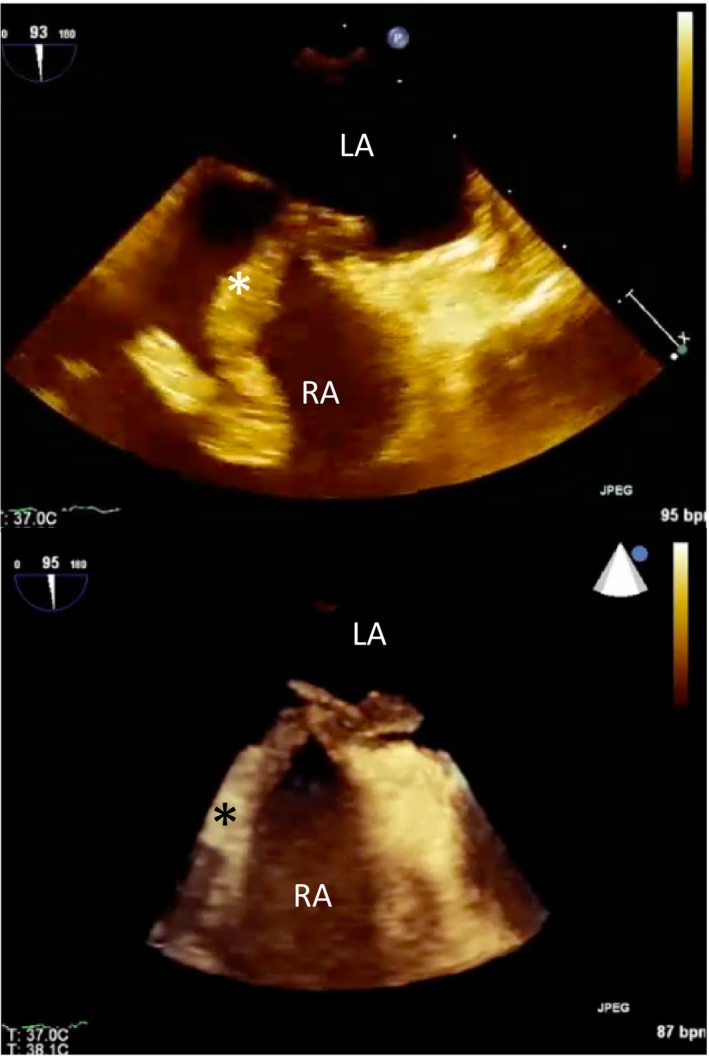
Clot in transit. Transesophageal echocardiogram images (2D and 3D) showing a large thrombus (*) in the right atrium (RA) traversing a patent foramen ovale (PFO) into the left atrium (LA). The patient suffered a fatal stroke.

The foramen ovale is an obligatory channel during fetal life that allows placental oxygenated blood to reach the arterial circulation of the fetus. When there is incomplete postnatal fusion of the septum primum and secundum, a PFO is formed.^9^ The presence of a PFO with either transient or continuous right‐to‐left shunt can potentially lead to paradoxical embolism. The PFO serves as a potential conduit for venous emboli to cross into the left atrium and eventually to the arterial circulation (Figure 1). The presence of an atrial septal aneurysm (ASA) has also been associated with cryptogenic stroke.^10^ An ASA is described as redundant bulging atrial septal tissue that can be caused by sustained interatrial pressure difference, or can be a primary malformation involving either the fossa ovalis or the entire atrial septum. It is objectively defined on echocardiography as 15‐mm of total septal tissue excursion or a 10‐mm protrusion into either atrium from the septal midline.^11^ Several studies have linked the presence of ASA to stroke. In 1 study, ASA was significantly more common in patients with stroke than in those without stroke (20 of 133 [15%] versus 12 of 277 [4%]; *P*<0.05).^12^ The combination of a PFO and ASA has been shown to be a significant risk factor for recurrent stroke.^10^

**Figure 2:**
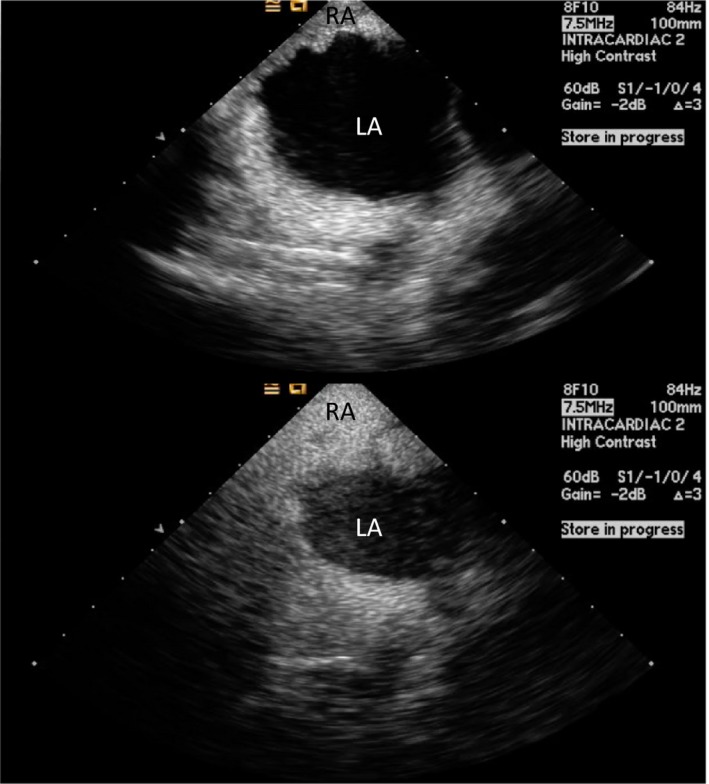
Intracardiac echo (ICE) showing a positive “bubble study” confirming a right‐to‐left shunt across a patent foramen ovale (PFO). Agitated saline is injected intravenously and bubbles are seen opacifying the right atrium (RA). Subsequently, bubbles are seen in the left atrium (LA) in less than 3 cardiac cycles.

**Figure 3:**
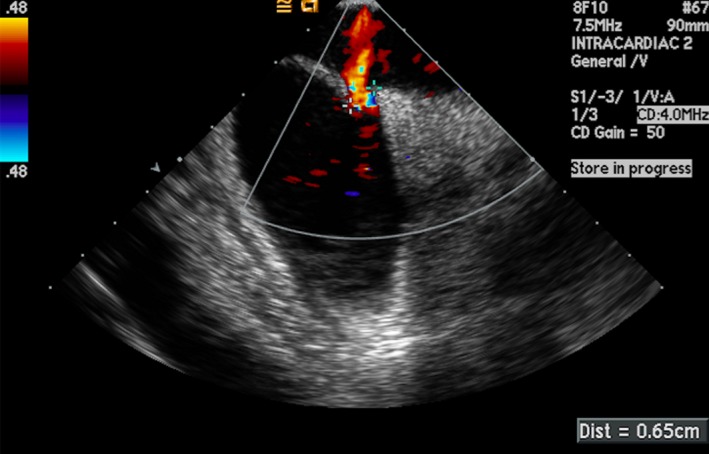
Intracardiac echocardiogram (ICE) image showing a PFO measuring 6.5 mm using color Doppler.

Evaluation of patients with suspected PFO should start with a transthoracic echocardiogram (TTE) and administration of agitated saline bubbles.^13^ The “bubble study” is best done in the standard apical 4‐chamber view. The timing of bubbles appearing into the left atrium is important to differentiate intracardiac from transpulmonary shunts. Intracardiac shunting is likely when bubbles appear in the left‐sided cardiac chambers within 3 cardiac cycles (Figure 2). There is no uniformly accepted grading scheme for assessing the degree of right‐to‐left shunting. However, Rana et al described a practical methodology: grade 1, less than 5 bubbles; grade 2, 5 to 25 bubbles; grade 3, more than 25 bubbles; and grade 4, opacification of the entire chamber.^11^ The bubble study is often done at rest and during a period of increased right heart pressure. This can be achieved during the release phase of the Valsalva maneuver when the right atrial pressure exceeds the left atrial pressure. Other techniques, such as coughing, sniffing, or applying manual external abdominal pressure, may also be used. Color Doppler should also be used to evaluate the presence of flow across the PFO, but this method is less sensitive for detecting small PFOs (Figure 3).^13^

TTE can also identify important morphological features of the PFO, such as the presence of an ASA, and quantify flow through the shunt. TTE remains the preferred initial diagnostic modality for detection of PFO; however, a transesophageal echocardiogram (TEE) or intracardiac echocardiography (ICE) is sometimes required to identify or further characterize the PFO and interatrial septum (IAS).^13^ TEE can clearly identify the anatomical detail of the PFO, including tunnel length, margins or rims, and surrounding structures (aorta, superior and inferior vena cava, pulmonary veins, and coronary sinus).^13^

Another method for detecting shunting from a PFO is transcranial Doppler (TCD) ultrasonography.^13^, ^14^ This method uses spectral Doppler frequency. A gaseous contrast agent, such as agitated saline, is injected in a peripheral vein, and the presence of microbubbles can be inferred by TCD recording in the middle cerebral artery as microembolic signals. Comparing TEE as the “gold standard,” TCD detection of PFO has a sensitivity of 95% and specificity of 75%.^15^ The test is deemed positive if the appearance of at least 1 bubble is recorded on the TCD trace within 40 seconds of injection. Quantification of right‐to‐left shunt by TCD is classified into: negative, no microbubbles; low‐grade shunt, 1 to 10 microbubbles; medium grade shunt, >10 microbubbles; and high grade shunt, “curtain effect” or numerous microbubbles recorded.^14^ This procedure is well tolerated by patients and can be done at bedside. Compared with TEE, advantages of TCD include increased patient comfort, a semiquantitative assessment of shunt size, and ability to detect intra‐ and extracardiac shunting.^13^

## History of PFO Closure

Percutaneous PFO closure techniques have been derived from established atrial septal defect (ASD) closure techniques. Surgical closure paved the way for minimally invasive or percutaneous closure techniques of both ASD and PFO. In 1947, Cohn reportedly closed ASDs in dogs using an atrial wall invagination technique.^16^ In Lyon, France, Santy performed the first successful closure of an ASD using right atrial appendage inversion in 1949. Shortly thereafter, in a series of experimental surgical closures, Hufnagel and Gillepsie used 2 nylon buttons through a right atriotomy in dogs,^17^ a technique that incurred a 100% mortality rate when later performed in 3 patients. The development of cardiopulmonary bypass technology opened the door for the rapid evolution of open cardiac surgical procedures. On May 6, 1953, John Gibbon successfully repaired an ASD in an 18‐year‐old woman while on cardiopulmonary bypass.^18^ After more than 3 decades of success with surgical closure, less‐invasive nonsurgical approaches for ASD and PFO closure were developed. In 1972, King and Mills developed an umbrella‐like device (King–Mills Cardiac Umbrella) for nonsurgical treatment of an ASD. It was composed of 6 stainless‐steel struts that contained fixation barbs and a Dacron covering for each opposing umbrella. The device was used to close 5 of 13 dogs with experimentally created ASDs.^19^ A 35‐mm King–Mills Cardiac Umbrella was then successfully implanted in a 17‐year‐old girl in 1975.^20^ Since then, there have been many versions of percutaneous ASD closure devices. The Gore Cardioform Septal Occluder (W.L. Gore and Associates, Inc,Newark, DE), and the Amplatzer PFO Occluder (Abbott Structural, Santa Clara, CA) have similar designs, with 2 opposing discs connected by a thin waist. Oppositional forces created by the 2 opposing discs seal the PFO. Both the Amplatzer PFO Occluder and the Gore Cardioform Septal Occluder are the only devices currently approved by the US Food and Drug Administration (FDA) for PFO closure in the United States; other devices are in the pipeline and awaiting future approval.

## FDA Approval Process

**Figure 4:**
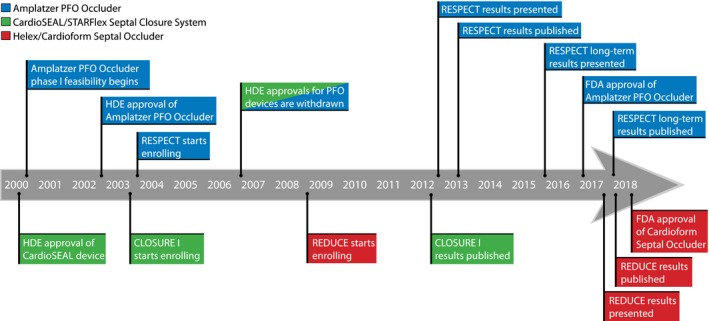
Timeline showing important dates of patent foramen ovale (PFO) closure trials and US Food and Drug Administration (FDA) milestones in the United States. HDE indicates Humanitarian Device Exemption.

As shown in Figure 4, the approval process for PFO closure device has spanned 2 decades. Before 2006, the use of percutaneous PFO closure in the US was only permitted under FDA Humanitarian Device Exemption for recurrent cryptogenic stroke from a PFO after failed conventional medical therapy. In 2006, the number of eligible patients exceeded the regulatory mandated annual limit of 4000 patients, and the Humanitarian Device Exemption process was voluntarily withdrawn. Randomized clinical trials for several devices were initiated and primarily focused on 3 devices: the Amplatzer PFO Occluder, the Starflex Septal Occluder (NMT Medical Inc, Boston, MA); and the Gore Cardioform Septal Occluder. These clinical trials have now all been completed and published (see Clinical Trials section below). Based on extended follow‐up results of the RESPECT and REDUCE trials, the FDA approved the Amplatzer PFO Occluder on October 28, 2016 and the Gore Cardioform Septal Occluder on March 30, 2018 for PFO closure in the United States *“*to reduce the risk of recurrent ischemic stroke in patients, predominantly between the ages of 18 and 60 years, who have had a cryptogenic stroke due to a presumed paradoxical embolism, as determined by a neurologist and cardiologist following an evaluation to exclude known causes of ischemic stroke.*”*

## The Amplatzer PFO Occluder

**Figure 5:**
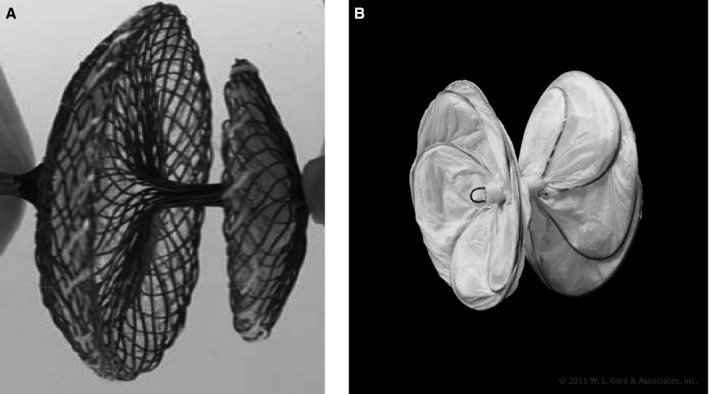
A, The Amplatzer PFO Occluder (courtesy of Abbott. ©2018 Abbott. All rights reserved) and B, the Gore Cardioform Septal Occluder (courtesy of W.L. Gore and Associates, Inc).

**Figure 6:**
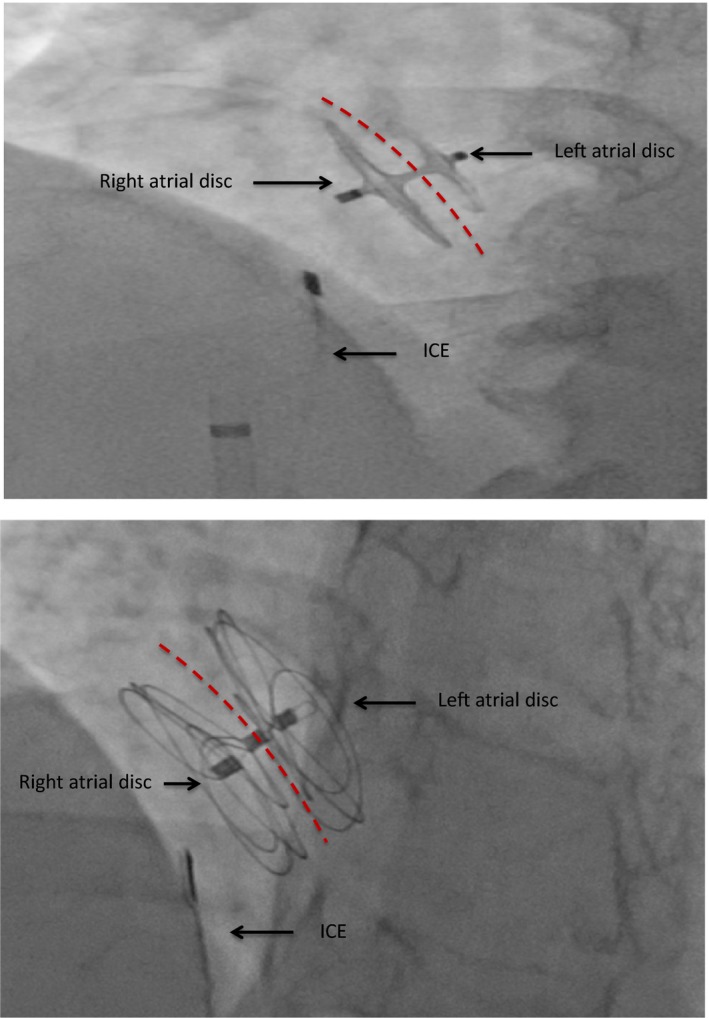
Fluoroscopic image of an Amplatzer PFO Occluder (top) and Gore Cardioform Septal Occluder (bottom) demonstrating a stable position in the atrial septum after release. The intracardiac echo (ICE) is seen in the right atrium. Red dotted lines represent the interatrial septum in this anteroposterior view.

Although there are several commercially available ASD and PFO closure devices on the market worldwide, the Amplatzer PFO Occluder was the first device to be FDA approved in the United States for PFO closure. The device has 2 self‐expanding discs composed of a nickel‐titanium (Nitinol) wire mesh with a wire diameter of 0.005 to 0.006 inches (Figure 5A). The wire mesh contains a polyester fabric that enhances the device's ability to seal the PFO and eliminate interatrial shunting. The 2 discs are connected by a short and thin waist (2 mm in diameter and 4 mm in length) that spans the PFO tunnel. Each disc is designed to conform to either side of the atrial septum and is available in 3 sizes (Figure 6). The device is most often delivered using the proprietary Amplatzer TorqVue delivery system that includes a sheath, dilator, delivery cable, and loader.

## The Gore Cardioform Septal Occluder

The Gore Cardioform Septal Occluder is currently FDA approved for both ASD and PFO closure. The device is also composed of 2 discs formed by platinum‐filled Nitinol wire frames covered by a proprietary thromboresistant expanded polyetrafluoroethylene material (Figure 5B). A 0.035‐ or 0.018‐inch guidewire may be used to advance the delivery system into the left atrium. The device was designed to be soft and conformable to surrounding anatomy of the atrial septum with minimal injury (Figure 6). It is available in a variety of sizes and comes preloaded in its own delivery system.

## PFO Closure for Recurrent Cryptogenic Stroke Prevention

### Randomized Clinical Trials Results

#### CLOSURE I Trial

The CLOSURE I (Evaluation of the STARFlex Closure System in Patients with a Stroke and/or Transient Ischemic Attack due to Presumed Paradoxical Embolism Through a Patent Foramen Ovale) trial was the first multicenter, open‐label, randomized trial of PFO closure for stroke prevention in the United States^21^ (Table 1). The trial was completed in 2008 and published in 2012. The trial randomized 909 patients in the United States and Canada to medical therapy consisting of aspirin alone or aspirin and warfarin versus closure with the STARFlex Septal Occluder and medical therapy. The primary end point was a composite of stroke or transient ischemic attack at 2 years, death from any cause within 30 days, or neurological death between 31 days and 2 years. The primary end point was observed in 5.5% in the closure group versus 6.8% in the medical therapy group (hazard ratio [HR], 0.78; 95% confidence interval [CI], 0.45–1.35; *P*=0.37).^21^ The trial concluded that in patients with cryptogenic stroke or transient ischemic attack who had a PFO, closure with this device did not have significant benefit compared with medical therapy in preventing recurrent stroke or transient ischemic attack. A major concern in the trial was that nearly half of the strokes in the closure group occurred within the first 30 days, suggesting that these events could have been related to device placement. Although effective closure, defined as procedural success with grade 0 or 1 residual shunt, was maintained in 86.7% of patients at 2‐year follow‐up, left atrial thrombus formation was noted in 1.1% of patients. Additionally, atrial fibrillation (AF) was reported in 5.7% of patients following PFO closure, potentially mitigating the effect of device closure. Enrollment in the trial was also hindered by the preference of some physicians and patients for percutaneous closure device leading them to decline participation in the trial. Because of real and perceived flaws in the design of the STARFlex device, it is no longer manufactured.

**Table 1:** Contemporary Randomized Trials on Percutaneous Closure of Patent Foramen Ovale

Trial Name	Year Published	PFO Device Used	Control Arm(s)	N	Mean Follow‐up (y)	Primary Endpoint	Results			Conclusions
							Closure	Control	*P* Value	
CLOSURE I	2012	STARFlex	Aspirin and/or Warfarin (INR 2–3)	909	2	Composite of stroke/TIA, all‐cause mortality, death from neurological causes	5.5%	6.8%	HR 0.78 95% CI 0.45 to 1.35 *P*=0.37	Closure is not superior to medical therapy
PC Trial	2013	Amplatzer PFO Occluder	Antiplatelet therapy or oral anticoagulation	414	4.1	Composite of death, nonfatal stroke, TIA, or peripheral embolism	3.4%	5.2%	HR 0.63 95% CI 0.24 to 1.72 *P*=0.34	Closure is not superior to medical therapy
RESPECT	2013	Amplatzer PFO Occluder	Aspirin or warfarin or Clopidogrel, or Aspirin with extended release dipyridamole	980	2.6	Composite of recurrent nonfatal ischemic stroke, fatal ischemic stroke, or early death after randomization	*Intention‐to‐treat* 0.66 events per 100 patients/year *As‐treated* 0.39 events per 100 patients/year	*Intention‐to‐treat* 1.38 events per 100 patients/year As treated 1.45 events per 100 patients/year	HR 0.49 95% CI 0.22 to 1.11 *P*=0.08 HR, 0.27 95% CI 0.10 to 0.75 *P*=0.007	No significant benefit for closure (intention‐to treat‐analysis) Closure is superior to medical therapy (as‐treated analysis)
RESPECT (Long‐term follow‐up)	2017	Amplatzer PFO Occluder	Aspirin or Warfarin or Clopidogrel, or Aspirin with extended release dipyridamole	980	5.9^a^	Composite of recurrent nonfatal ischemic stroke, fatal ischemic stroke, or early death after randomization	*Intention‐to‐treat* 0.58 events per 100 patients/year *New stroke of unknown mechanism* 0.31 events per 100 patients/year	*Intention‐to‐treat* 1.07 events per 100 patients/year *New stroke of unknown mechanism* 0.86 events per 100 patients/year	HR 0.55 95% CI 0.31 to 1.0 *P*=0.046 HR 0.38 95% CI 0.18 to 0.79 *P*=0.007	Closure is superior to medical therapy on extended follow‐up in intention‐to‐treat analysis
CLOSE	2017	Any CE marked PFO device	1) *Antiplatelet arm*: Aspirin or Clopidogrel or Aspirin with extended release dipyridamole 2) *Oral anticoagulant arm*: Vitamin K antagonists or NOACs	663	5.3	Recurrent fatal or nonfatal stroke	*Closure vs antiplatelet therapy:* 0	*Closure vs antiplatelet therapy* 4.9% 5‐year estimate *Anticoagulant vs Antiplatelet therapy* 1.5% vs 3.8%, respectively, 5‐year estimate	*Closure vs antiplatelet therapy* HR 0.03 95% CI 0 to 0.26 *P*<0.001 *Anticoagulant vs Antiplatelet therapy* HR 0.43 95% CI 0.1 to 1.5 *P*=0.17	Closure is superior to antiplatelet in patients with ASA or PFO with large shunt Anticoagulant is equivalent to antiplatelet therapy
REDUCE	2017	Helex Septal Occluder and Cardioform Septal Occluder	Aspirin or Clopidogrel or Aspirin with dipyridamole	664	3.2^a^	1) Recurrent stroke 2) New brain infarct inclusive of silent brain infarct (SBI)	*Ischemic stroke:* 1.4% *New brain infarct:* 5.7%	*Ischemic stroke:* 5.4% *New brain infarct:* 11.3%	HR 0.23 95% CI 0.09 to 0.62 *P*=0.002 HR 0.51 95% CI 0.29 to 0.91 *P*=0.04	Closure is superior to antiplatelet therapy
DEFENSE‐PFO	2018	Amplatzer PFO Occluder	Aspirin or Aspirin and Clopidogrel, or Aspirin and Cilostazol, or Warfarin	120	2.8^a^	Stroke, vascular death or TIMI‐defined major bleeding	*Ischemic stroke:* 0 *2 year event rate:* 0 *New ischemic lesion on MRI:* 8.8%	*Ischemic stroke:* 10.5% *2 year event rate:* 12.9% *New ischemic lesion on MRI:* 18.4%	*P*=0.023 Log‐rank *P*=0.013 *P*=0.24	Closure in patients with high risk PFO characteristics resulted in lower rate of ischemic stroke versus medical therapy

ASA indicates atrial septal aneurysm; CI, confidence interval; CLOSE, Closure of Patent Foramen Ovale or Anticoagulants Versus Antiplatelet Therapy to Prevent Stroke Recurrence; CLOSURE I, Evaluation of the STARFlex Septal Closure System in Patients with a Stroke and/or Transient Ischemic Attack Due to Presumed Paradoxical Embolism through a Patent Foramen Ovale; DEFENSE‐PFO, Device Closure Versus Medical Therapy for Cryptogenic Stroke Patients With High‐Risk Patent Foramen Ovale; HR, hazard ratio; INR, international normalized ratio; N, number of patients; NOACs, novel oral anticoagulants; PC, Percutaneous Closure of Patent Foramen Ovale Using the AMPLATZER PFO Occluder with Medical Treatment in Patients with Cryptogenic Embolism; PFO, patent foramen ovale; REDUCE, GORE HELEX Septal Occluder/GORE CARDIOFORM Septal Occluder and Antiplatelet Medical Management for Reduction of Recurrent Stroke or Imaging‐Confirmed TIA in Patients With Patent Foramen Ovale (PFO); RESPECT, Randomized Evaluation of Recurrent Stroke Comparing PFO Closure to Established Current Standard of Care Treatment; TIA, transient ischemic attack; TIMI, thrombolysis in myocardial infarction.

a Median follow‐up reported.

#### PC Trial

The PC (Clinical Trial Comparing Percutaneous Closure of Foramen Ovale Using the Amplatzer PFO Occluder with Medical Treatment in Patients with Cryptogenic Embolism) trial was conducted in 29 sites in Europe, Canada, Brazil, and Australia and published in 2013.^22^ The trial compared device closure with Amplatzer PFO Occluder versus best medical therapy in patients aged <60 years who had a PFO and an ischemic stroke, transient ischemic attack, or a peripheral thromboembolic event. The trial ultimately enrolled a total of 414 patients (Table 1). The primary end point, which was similar to the CLOSURE I trial, occurred in 7 patients in the closure group and 11 in the medical therapy group (HR, 0.63; 95% CI, 0.24–1.62: *P*=0.34).^22^ Although fewer strokes occurred in the closure group, the results, once again, did not reach statistical significance.

The CLOSURE I and PC trials had similar results with a nonstatistically significant trend toward benefit for closure device as secondary prevention of stroke compared with current medical therapy. Both trials, however, did not achieve the prespecified clinical end point for efficacy. The impact of these 2 “negative” trials on PFO closure in the United States was profound, and for years the procedure was largely forgotten and not supported by stakeholder societies and third‐party payers.

#### RESPECT Trial

The landmark study, RESPECT (Randomized Evaluation of Recurrent Stroke Comparing PFO Closure to Established Current Standard of Care Treatment) trial compared medical therapy with 1 or more antiplatelet medications or warfarin alone with PFO closure using the Amplatzer PFO Occluder in 980 patients with cryptogenic stroke (Table 1).^23^ The primary efficacy end point was nonfatal ischemic stroke, fatal ischemic stroke, or early death after randomization. With a mean follow‐up of 2.6 years, the intention‐to‐treat cohort demonstrated a recurrence of stroke in 9 patients in the closure group and 16 in the medical therapy group (HR, 0.49; 95% CI, 0.22–1.11; *P*=0.08). Of note, 3 of the 9 patients in the closure group had a stroke without a device in place. One patient had a stroke after randomization before a closure device was placed, the second patient decided not to proceed after the stroke, and the third had a stroke during an unexpected coronary artery bypass graft surgery wherein the PFO was closed surgically. However, in the prespecified per‐protocol cohort, 6 patients in the closure group and 14 in the medical therapy group had a recurrent stroke (HR, 0.37; 95% CI, 0.14–0.96; *P*=0.03).^23^ Although the intention‐to‐treat cohort did not reach significance for the efficacy end point, both the prespecified per‐protocol and as‐treated analyses (5 events in the closure group versus 16 in the medical therapy group) suggested superiority of closure over medical therapy. The FDA requested supplemental long‐term analysis of the RESPECT patient cohort before considering approval of the device for PFO closure. Subsequently, in October 2015, the RESPECT investigators presented the results from their long‐term patient follow‐up (mean of 5.9 years).^24^ The intention‐to‐treat analysis now demonstrated a significant reduction in recurrent ischemic strokes in the PFO closure arm (HR, 0.55; 95% CI, 0.305–1.0; *P*=0.046).^24^ Reduction in new stroke of unknown mechanism was significant in the closure arm and superior to medical therapy (HR, 0.38; 95% CI, 0.18–0.79; *P*=0.007). Both groups reported similar rates of AF (0.25 per 100 patient‐years versus 0.15 per 100 patient‐years; *P*=0.37), and no device embolization or erosion were reported in the trial. The closure group did experience a higher number of deep venous thrombosis (1% versus 0.2%; *P*=0.218) and pulmonary embolism (2.4% versus 0.6%; *P*=0.034), which may be explained by a higher use of warfarin therapy in the medical group.^25^ Final results were published on September 14, 2017.^25^ Results of the long‐term follow‐up of the RESPECT trial led to FDA approval of the Amplatzer PFO Occluder device.

#### REDUCE Trial

On August 19, 2008, The Gore‐REDUCE (Gore Helex Septal Occluder/Gore Septal Occluder for Patent Foramen Ovale Closure in Stroke Patients) clinical study began enrollment of patients aged 18 to 59 years who had a cryptogenic ischemic stroke within 180 days of randomization. A total of 664 patients were randomized either to antiplatelet therapy alone or PFO closure with the Helex Septal Occluder device or with the Cardioform Septal Occluder device, plus antiplatelet therapy. Because of design refinements, the Helex Septal Occluder device was replaced in late 2012 by the Cardioform Septal Occluder device. The co‐primary end points were freedom from recurrent ischemic stroke or new silent brain infarct on imaging. The final results demonstrated a statistically significant reduction for both primary end points. Clinical ischemic stroke occurred in 1.4% in the closure group and 5.4% in the medical therapy group (HR, 0.23; 95% CI, 0.09–0.62; *P*=0.04; Table 1).^26^ New brain infarctions were also noted to be significantly lower in the closure group (5.7%) than in the medical therapy group (11.3%; relative risk, 0.51; 95% CI, 0.29–0.91; *P*=0.04).^26^ There was a significantly higher rate of AF reported in the device closure arm, which was mostly periprocedural and transient (6.6% versus 0.4%; *P*≤0.001).^26^ The robust clinical results of the REDUCE trial led to FDA approval of the Gore Cardioform Septal Occluder for PFO closure on March 30, 2018.

#### CLOSE Trial

The CLOSE (Closure of Patent Foramen Ovale, Oral Anticoagulants or Antiplatelet Therapy to Prevent Stroke Recurrence) trial is a multicenter, open‐label, randomized, 3‐group superiority trial conducted at 32 sites in France and 2 sites in Germany.^27^ Compared with other contemporary trials for PFO closure, the CLOSE trial was unique in 2 ways. First, it had 3 groups that included: (1) the device group; (2) antiplatelet or antithrombotic group; and (3) an oral anticoagulant group. Anticoagulants used in the study were comprised of vitamin K antagonists and novel oral anticoagulants. Second, the study used any PFO closure device with a CE mark and approved by the Interventional Cardiology Committee. The CLOSE trial investigated whether PFO closure in addition to antiplatelet therapy was superior to antiplatelet or anticoagulant therapy alone in preventing stroke recurrence. They included patients aged 16 to 60 years who had been diagnosed with a previous cryptogenic stroke and either an ASA or a large right‐to‐left shunt (more than 30 microbubbles in the left atrium within 3 cardiac cycles). The primary outcome was fatal or nonfatal stroke. A total of 663 patients were evaluated with a mean follow‐up of 5.3 years (Table 1).

The CLOSE trial revealed that PFO closure significantly reduced the risk of recurrent stroke compared with antiplatelet therapy (HR, 0.03; 95% CI, 0–0.26; *P*<0.001; Table 1).^27^ The anticoagulant group, although showing a trend toward superiority, did not show a significant benefit over antiplatelet therapy alone (HR, 0.43; 95% CI, 0.1–1.5; *P*=0.17). A notable observation in the study is again the increased risk of AF with PFO closure during the periprocedural period compared with antiplatelet therapy alone (4.6% vs. 0.9%; *P*=0.02).^27^

#### DEFENSE‐PFO Trial

The DEFENSE‐PFO (Device Closure Versus Medical Therapy for Cryptogenic Stroke Patients With High‐Risk Patent Foramen Ovale) trial was a multicenter, randomized, open‐label, superiority trial carried out in Korea that compared percutaneous PFO closure using the Amplatzer PFO Occluder to medical therapy in patients with cryptogenic stroke and high‐risk PFO.^28^ This trial was different from previous trials and enrolled only patients with high‐risk morphological features defined as those patients with an ASA, hypermobility of the IAS, or a large‐sized PFO (≥2 mm). Interestingly, primary endpoint events consisting of stroke, vascular death, or Thrombolysis In Myocardial Infarction–defined major bleeding occurred exclusively in the medical therapy–only group (2‐year event, 12.9% [log‐rank, *P*=0.013]; Table 1). Interim analysis of results led to premature cessation of the trial because of the observed differences in outcomes of the treatment arms and the overwhelmingly positive results of the other randomized clinical trials favoring device closure. Results of the study suggest that the benefits of PFO closure may be, in part, based on morphological characteristics (ie, high‐risk features) of the PFO, such as presence of ASA, hypermobility of the IAS, and large‐sized PFOs.

## Other Potential Indications for PFO Closure

### Migraine With Aura

Several retrospective, observational studies have noted improvement in migraine headaches in patients who have undergone PFO closure for nonmigraine indications.^29^ A causal relationship between right‐to‐left shunting across a PFO and migraine headache has been proposed, but remains unproven. Epidemiological studies have shown that 48% of patients who experience migraine with aura (but not migraine without aura) had a PFO.^30^ Several theories have been proposed to explain this association. The presence of a persistent right‐to‐left PFO‐mediated shunt allows for passage of vasoactive amines and humoral substances that are normally metabolized, activated, and inactivated by the lungs. Such substances include prostaglandin E1, E2, serotonin, bradykinin, and angiotensin I. In the presence of a right‐to‐left shunt, these substances reach the cerebrovascular circulation bypassing the lungs, triggering a migraine with aura. A second theory proposes that microthrombi or emboli from the venous circulation pass through the PFO and enter the posterior circulation. Support for this notion is derived from the observation that the occipital lobe is the predominant region of infarct in patients suffering from migraine with aura.^31^

The impact of PFO closure on migraine symptoms has been studied in few randomized clinical trials. The MIST (Migraine Intervention with STARFlex Technology) trial was a prospective, multicenter, double‐blind, sham‐controlled trial conducted in the United Kingdom, which aimed to evaluate the efficacy of PFO closure with the STARFlex device in patients with moderate‐to‐large right‐to‐left shunt and refractory migraine headaches. The trial showed no significant difference in the primary end point of migraine cessation between the implant and sham group.^32^ A follow‐up trial, the MIST II trial, was conducted in the United States and was terminated shortly after initiation because of slow enrollment. The PRIMA (Percutaneous Closure of PFO in Migraine with Aura) trial also randomized patients to investigate the effectiveness of PFO closure in patients suffering from migraine with aura. An Amplatzer PFO Occluder was implanted in 53 patients (closure group) whereas 54 received medical management (control group). The trial was also prematurely terminated because of slow enrollment. The study concluded that the PFO closure group did not reduce monthly migraine days.^33^ Recently, The PREMIUM (Prospective, Randomized Investigation to Evaluate Incidence of Headache Reduction in Subjects With Migraine and PFO Using the Amplatzer PFO Occluder to Medical Management) trial was published.^34^ It is a double‐blind, sham‐controlled trial that investigated migraine attacks over 1 year in subjects with PFO closure with the Amplatzer PFO Occluder and medical therapy versus medical therapy with sham procedure. The recently published results showed that PFO closure did not meet the primary end point of 50 percent or greater reduction in migraine attacks compared with sham control. The relationship between PFO and migraine headache and benefits of device closure remains unclear, and further data from clinical trials are needed.

### Decompression Sickness

Decompression sickness (DCS) is another potential application of PFO closure. DCS occurs when individuals are exposed to elevated nitrogen pressure when breathing compressed air using Self‐Contained Underwater Breathing Apparatus (SCUBA) during deep water diving. During rapid ascent, dissolved nitrogen in the body becomes supersaturated and undergoes vascular and extravascular bubble formation. These bubbles can then cause pulmonary DCS characterized by pain, cough, and dyspnea as gas bubbles accumulate in the pulmonary capillaries. In patients with PFO, paradoxical embolism of bubbles can cause neurological and cutaneous DCS symptoms. The association between PFO and DCS was described by Moon in 1989 using echocardiography and Doppler in divers.^35^ The risk of DCS in divers with a PFO is 5 times higher than divers without one. The risk is also proportional to the size of the PFO.^36^ A case‐control study examined 47 divers with unprovoked DCS and a documented PFO and assigned half of them to PFO closure (the other half was used as control). In the PFO closure group, 5 (25%) were treated with the Amplatzer Septal Occluder and 15 (75%) with the Occlutech Figulla PFO Occluder N (Occlutech GmbH, Jena, Germany). Using TTE to detect venous bubbles and transcranial color‐coded sonography to detect arterial bubbles, the researchers found no difference in the appearance of venous bubbles in both groups after a test dive. Interestingly, catheter‐based PFO closure led to complete elimination of arterial bubbles.^37^ None of the divers in either group suffered DCS. Suggested recommendations for divers with diagnosed PFO and a history of DCS include the cessation of diving, a conservative approach to diving, and consideration for PFO closure. Further randomized clinical trials are required to evaluate the benefit of PFO closure in these patients.

### Platypnea‐Orthodeoxia Syndrome

Platypnea‐orthodeoxia syndrome (POS) is an uncommon and often underdiagnosed conundrum described as positional dyspnea (platypnea) and hypoxemia (orthodeoxia). Symptoms of POS are exacerbated during the upright position, but improve when recumbent.^38^ Although the presence of a right‐to‐left shunt through a PFO is the suspected mechanism, hypoxemia can also be caused by pulmonary shunting or ventilation‐perfusion mismatch. The upright position changes the conformation of an existing interatrial communication, thereby increasing the right‐to‐left shunt and worsening hypoxemia. The orientation of the inferior vena cava (IVC) with the foramen ovale, when recumbent, decreases the right‐to‐left shunt. Several case reports have been published describing the improvement of POS following PFO closure. Sorrentino described the case of a patient with a PFO and POS in 1991. The patient reported significant shortness of breath when sitting up and was found to have a right‐to‐left shunt when lying supine, which increased when seated upright. The PFO was closed surgically and the patient's symptoms quickly resolved.^39^ Several other case reports indicate successful correction of the hypoxemia and symptoms after PFO closure.^38^, ^40^, ^41^ Mojadidi et al prospectively studied 683 patients with PFO‐associated conditions, of which 17 (2.5%) had POS and had elected to close their PFO. Improved oxygen saturation was noted in 11 of 17 patients (64.8%) as well as improvement or complete resolution of symptoms. Patients who had no improvement after PFO closure predominantly had a pulmonary cause for their hypoxia.^42^ There is a paucity of data regarding the potential benefit of PFO closure for POS, but closure could be considered in cases of severe hypoxia after pulmonary disease has been excluded.

### PFO in Patients Undergoing Liver Transplantation

Paradoxical embolism is a well‐described phenomenon in the setting of liver transplantation. The presence of a PFO has been implicated in both air embolism and thromboembolism during or immediately following surgery.^43^, ^44^ There is concern that a PFO may be a conduit for air embolism from placement of central lines, veno‐venous bypass, caval clamping, or inadequate flushing of the harvested transplant. The possibility of PFO‐mediated paradoxical embolism has stimulated interest among transplant centers in treating PFO with device closure before liver transplant surgery. This application has not been supported by randomized clinical trial data. Retrospective studies of liver transplant patients with existing PFO did not demonstrate improvement in length of stay in the intensive care unit, postoperative oxygen requirements, nor 30‐day mortality when compared with similar patients without PFO.^45^, ^46^ Cerebrovascular accidents were not observed in either group. The conclusion was that the presence of a PFO does not adversely impact clinical outcomes after liver transplant surgery and that routine PFO closure should not be recommended for patients before surgery.

## Patient Selection

### Cryptogenic Stroke Evaluation; Which PFO Should We Close?

Evidence supporting PFO‐mediated paradoxical embolic stroke include: cortical location of infarcts, strokes in multiple vascular distributions, and infarcts of different ages in the same vascular territory.^47^ A presumptive diagnosis of cryptogenic stroke can be inferred after all other causes of ischemic stroke have been eliminated. Other indirect evidence of a cryptogenic stroke would be absence of conventional stroke risk factors, a history of deep vein thrombosis, recent travel, pulmonary embolus, or Valsalva maneuver before the stroke event. Lacunar infarcts are generally not associated with embolic events.^48^ For the purposes of the randomized clinical trials, stroke was defined as an acute neurological deficit, presumably attributed to ischemia, that either resulted in clinical symptoms lasting 24 hours or longer, or symptoms lasting less than 24 hours but associated with a new, neuroanatomically relevant, cerebral infarct on noninvasive imaging.

The RoPE (Risk of Paradoxical Embolism) score has been developed and validated as an assessment tool to determine the probability that a PFO is responsible for a cryptogenic stroke.^49^, ^50^ It can be used when assessing patients with a PFO preceding closure (Table 2). A high score correlates with increased likelihood that a PFO is responsible for the index stroke. The PFO‐attributable fraction of stroke for a score of 7, 8, and 9 is 72%, 84%, and 88%, respectively, and defines a subset of patients who may benefit from PFO closure. The risk‐benefit ratio of performing PFO closure in patients with a RoPE score less than 7 should be carefully weighed.^49^

**Table 2:** RoPE Score

Patient Characteristic	Points
No history of hypertension	1
No history of diabetes mellitus	1
No history of TIA or stroke	1
Nonsmoker	1
Cortical infarct on imaging	1
Age, y	
18 to 29	5
30 to 39	4
40 to 49	3
50 to 59	2
60 to 69	1
>70	0

Reprinted from Kent et al^50^ with permission. Copyright ©2013, Wolters Kluwer Health, Inc. RoPE indicates Risk of Paradoxical Embolism; TIA, transient ischemic attack.

Before considering PFO closure, a careful evaluation should be done to rule out other causes of stroke, including hypercoagulable states, atherosclerotic lesions, other cardioembolic sources, and arterial dissection. Table 3 outlines a suggested diagnostic workup for a patient with suspected cryptogenic stroke.^51^ One of the most important conditions to exclude is AF. Occult AF has been identified in up to 16% of cryptogenic stroke within 90 days of randomization.^52^ Noninvasive electrocardiographic monitoring for 30 days improved detection of AF compared to 24‐hour monitoring.^52^ A period of extended cardiac monitoring should then be performed in every patient with a cryptogenic stroke before considering PFO closure. Unmasking occult AF or atrial flutter would suggest an etiological association and mandate guideline‐directed chronic anticoagulation as opposed to percutaneous PFO closure.

**Table 3:** Cryptogenic Stroke Workup

Condition	Recommended Testing
Hypercoagulable disorder	CBC (hemoglobin and platelet count), factor V Leiden, protein C, protein S, antithrombin III, homocysteine levels, prothrombin G20210A mutation, and antiphospholipid antibodies
Paroxysmal atrial fibrillation	≥30‐day continuous cardiac monitoring
Cardiac thrombus, vegetation, or tumor; mitral stenosis	TTE followed by TEE (if TTE is normal); cardiac CT or MRI can be considered if high suspicion
Carotid atherosclerotic disease	Carotid duplex ultrasound, CTA, or MRA of the neck and head
Cerebral vascular atherosclerotic disease	CTA or MRA of the head
Aortic arch atheroma	TEE or CTA of the chest
Arterial dissection	CTA of the chest and neck
Cerebral venous sinus thrombosis	Brain MRV
May–Thurner syndrome	Pelvic MRV

Reprinted from Poulin et al^51^ with permission. Copyright ©2017, Bryn Mawr Communications. CBC indicates complete blood count; CTA, computed tomography angiography; MRA, magnetic resonance angiography; MRV, magnetic resonance venography; TEE, transesophageal echocardiography; TTE, transthoracic echocardiography.

### Heart‐Brain Team Evaluation

The benefit of a patient‐centered, multidisciplinary team evaluation for structural heart disease procedures has already been demonstrated for transcatheter aortic valve replacement, percutaneous mitral valve repair, complex coronary artery disease interventions, and left atrial appendage (LAA) closure. FDA approval of the Amplatzer PFO Occluder and the Gore Cardioform Septal Occluder clearly mandates that patients be evaluated by both a cardiologist and a neurologist (ideally a stroke neurologist) before consideration of PFO closure. The heart‐brain team fosters a shared decision‐making process between the patient and a multidisciplinary team of physicians, ensures proper patient selection, serves to prevent inappropriate PFO closure, and mitigates unnecessary risks.

## Procedural Techniques

### Patient Preparation

The ideal antiplatelet regimen preceding PFO closure has not been studied. Most operators will pretreat patients with aspirin (81–325 mg) and/or clopidogrel (75 mg). In the CLOSE and RESPECT clinical trials, all patients received aspirin and clopidogrel on the day of the procedure, whereas only clopidogrel was administered in the REDUCE trial.^25^, ^26^, ^27^ Duration of dual antiplatelet therapy post PFO closure is discussed under Postprocedure Care below. Patients on chronic antithrombotic therapy for other indications who are high risk for thromboembolic events may be bridged at the physician's discretion. Other patients at lower risk should have their medication held before the procedure. Although data on antimicrobial prophylaxis for cardiac device implantation are scarce, administration of prophylactic antibiotic therapy within 60 minutes of the procedure start time using a first‐generation cephalosporin (such as cefazolin 2 g IV) is recommended.^53^

### Access Site

The femoral vein, preferably the right femoral vein, remains the most common approach for PFO closure. Location of the PFO within the IAS is well suited to the femoral approach, allowing for easier access of wires, catheters, and delivery sheaths. The presence of a filter device in the IVC does not usually preclude a femoral approach. Most IVC filters can easily be traversed with wires and catheters provided care is used to avoid dislodgement or entrapment within the filter device.^54^ However, in rare cases, the presence of an IVC occlusion may preclude a femoral venous approach. In these situations, alternative access sites, such as the internal jugular vein,^55^ axillary vein,^56^ and hepatic vein,^57^ have been successfully used for device delivery.

If an Amplatzer PFO occluder is used, an 8‐ or 9‐French femoral vein introducer sheath is placed. An 11‐French sheath is used if a Gore Cardioform Septal Occluder is chosen. If ICE is used for imaging, a second 8‐French sheath is placed either on the contralateral side or below the first introducer sheath on the ipsilateral side. Vascular injury with the rigid ICE catheter can be avoided by using a 23‐cm introducer sheath to traverse the bifurcation of the IVC. It is prudent to use fluoroscopic guidance when introducing and manipulating the ICE catheter.^58^ PFO procedures require anticoagulation, and intravenous unfractionated heparin is generally given after vascular access is achieved at a dose of 70 to 100 IU/kg to maintain an activated clotting time of more than 250 seconds.

### Imaging Guidance

**Figure 7:**
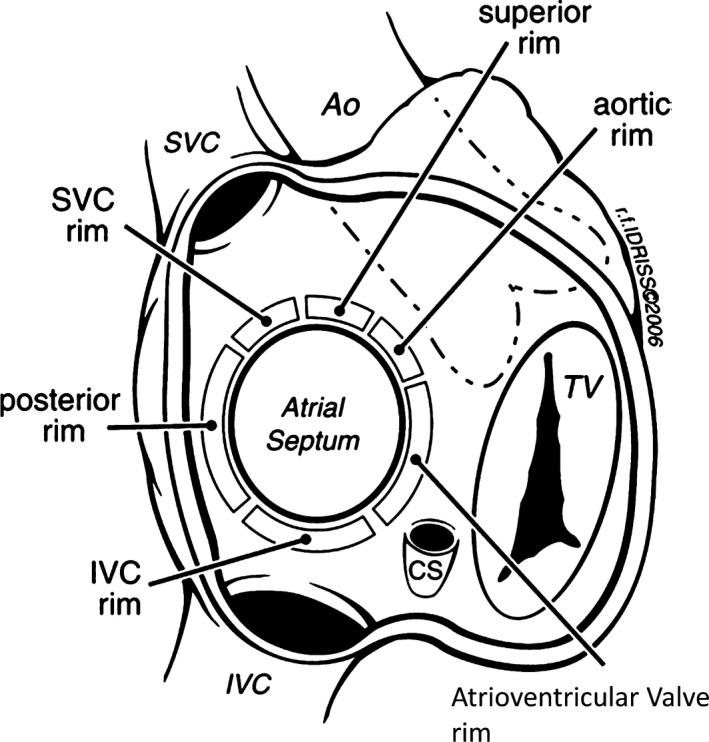
Rims of the atrial septum as seen from the right atrium. Ao indicates aorta; SVC, superior vena cava; IVC, inferior vena cava; TV, tricuspid valve. Reprinted from Amin et al^59^ with permission. Copyright ©2006, John Wiley & Sons.

High‐quality intraprocedural echocardiographic imaging is essential for successful PFO closure. Because of wide variations in the anatomy of the IAS, the atrial septum must be carefully interrogated to avoid complications and safely deploy the PFO closure device. Operators should be familiar with the anatomy of the atrial septum and its surrounding structures. Location of the aortic root and its close relationship to the septum should be recognized. The aortic root abuts the atrial septum, creating an in folding (transverse pericardial sinus). Avoidance of this area during guide wire and catheter advancement is paramount to avoid iatrogenic perforation into the transverse pericardial sinus or the aorta. As with ASD closure, detailed knowledge of relevant rims surrounding the atrial septum is important. The classification of the atrial septal rims, as proposed by Amin, include the following: aortic rim, superior vena cava (SVC) rim, superior rim (between the SVC rim and the aortic rim), the posterior rim (opposite the aortic rim), inferior vena cava (IVC) rim, and the atrioventricular rim (Figure 7).^59^

Several specific key findings on imaging are useful for the operator in planning for PFO closure. Fundamental characteristics include tunnel length, presence of ASA, thickness of the septum secundum, presence of additional defects, and presence of additional structures in the right atrium, such as a Eustachian valve or Chiari network. Absence of right and left atrial thrombi should also be confirmed before the procedure since that they would denote contraindications to the procedure.

**Figure 8:**
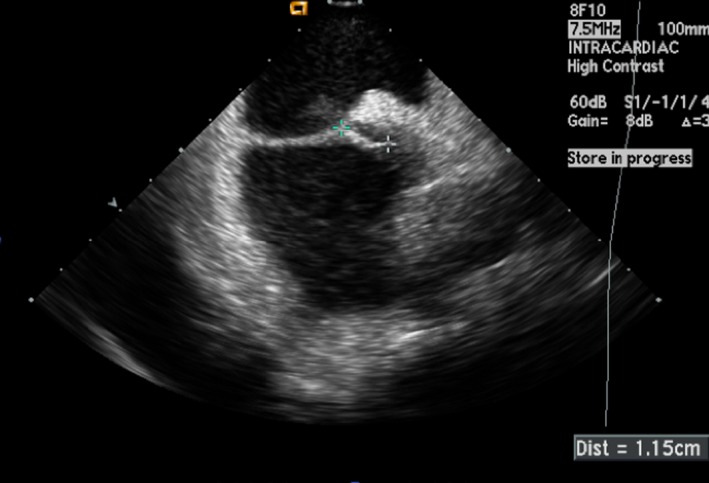
Intracardiac echocardiogram showing a patent foramen ovale (PFO) with a tunnel length of 1.15 cm.

Tunnel length is measured using TEE in the bicaval view or ICE (Figure 8). Presence of a long tunnel makes PFO closure technically more difficult. Although there is no defined length criterion for long PFO tunnel, a length of more than 12 mm has been arbitrarily used in some case reports.^60^ Inadequate disc apposition, residual shunts, and inability to deploy the device are possible difficulties encountered when treating a PFO with a long tunnel. There are case reports of transseptal puncture through the septum primum of the fossa ovalis near the septum secundum and the tunnel origin to facilitate device deployment in patients with long tunnels. This technique is, however, associated with high incidence of residual shunting.^61^ Measurement of the PFO tunnel width is also a crucial parameter to assess. A wide tunnel may also result in failure of closure or device embolization.^11^ In this situation, the operator may want to select a larger device.

The presence of an ASA has been associated with an incremental risk of cryptogenic stroke, embolic stroke, and larger PFO size.^62^ An ASA is also associated with increased risk of device embolization, and larger devices may be helpful in these situations to stabilize the aneurysmal portion of the septum.^63^

A thick septum secundum may prevent the device from conforming well to the septum, splaying out the right and left atrial discs, leading to a residual shunt. In this scenario, a softer, more‐compliant device may better conform to the thick septum secundum and prevent residual shunt.

Finally, the presence of pacemaker leads in the right atrium and right ventricle, prominent Eustachian valve, and Chiari network can make technical performance of the procedure challenging and should be taken into account during procedural planning.

#### TEE Versus ICE

The role of TEE during transcatheter ASD and PFO closure has been well established.^11^ TEE provides excellent image quality of the entire IAS, as well as adjacent cardiac and extracardiac structures. TEE can effectively image left atrial and LAA thrombi, prominent Eustachian valves and Chiari networks. With the echocardiographer as the main TEE operator, the proceduralist is freed from additional tasks during device closure. TEE almost always requires general anesthesia, which exposes the patient to risks of general anesthesia, esophageal trauma, aspiration, and patient discomfort. Relying on TEE for PFO closure requires the presence of an additional physician to manipulate the TEE probe and acquire necessary images.

In light of this, there has been a growing interest with the use of ICE during transcatheter procedures, specifically PFO and ASD closures. ICE imaging quality has been shown to be comparable to TEE and allows for a better visualization of the septal rims before closure.^13^, ^64^ ICE also obviates the need for general anesthesia. In addition, the operator has complete control over image acquisition, eliminating the need for an additional physician for the procedure. The additional cost of the ICE catheter can be balanced against the cost savings of avoiding general anesthesia, an anesthesiologist, and another physician for imaging.^65^, ^66^ ICE requires additional venous access (usually femoral) and is associated with rare complications, which include vascular injury, cardiac perforation, and atrial arrhythmias.^13^ When used by experienced operators, ICE can be performed safely and effectively as demonstrated in a large cohort of children and adolescents who underwent percutaneous device closure.^64^ ICE catheters currently available in the United States are the AcuNav Ultrasound Catheter (Siemens, Munich, Germany), ViewFlex Xtra Intracardiac Echocardiography catheter (St. Jude Medical, now Abbott), Ultra ICE Plus (Boston Scientific, Boston, MA), and Foresight Intracardiac Echocardiography System (Conavi Medical Inc, North York, ON, Canada) These ICE systems are available in 8‐ and 9‐French sizes. The AcuNav Ultrasound Catheter system is also available in 10‐French size. New ICE platforms feature 3‐dimensional imaging, such as the ACUSON AcuNav V ultrasound catheter.^67^

Regardless of imaging modality, a comprehensive echocardiographic evaluation with special attention to the anatomical features of the IAS and the surrounding structures is critical for the safe and effective placement of a PFO device.

### Crossing the PFO

The level of difficulty encountered when crossing a PFO from the right atrium is highly variable and depends on the tunnel length, tunnel width, presence of an ASA, prominent Eustachian valve, Chiari network, or pacemaker leads. The PFO can be crossed directly with a catheter such as a multipurpose catheter or a balloon‐tipped floatation catheter with the balloon deflated. Catheters are generally positioned in the SVC or brachiocephalic vein and then withdrawn into the heart, where a slow clockwise or posterior rotation will usually engage the fossa. Fluoroscopy and echocardiographic guidance used in combination are quite helpful in crossing the PFO. In small PFOs or long tunnels, difficulty may be encountered engaging and passing a catheter through the PFO.

Once the catheter has crossed the PFO, a left atrial pressure tracing should be seen. Left atrial blood saturation may be obtained to confirm catheter position. Catheter position in the left atrium is always confirmed by echocardiographic imaging. Gentle flushing of the catheter with heparinized saline is done, making sure that there are no clots or air in the system. Heparin should be administered, preferably at the beginning of the procedure, to achieve a therapeutic activated clotting time above 250 seconds and confirmed before to entering the left atrium.

A soft‐tip 0.035‐inch guidewire such as a Wholey wire (Covidien Ltd, Dublin, Ireland) or a Glidewire (Terumo Medical Corporation, Tokyo, Japan) is then introduced through the catheter. The wire and catheter assembly is then advanced and secured in the left upper pulmonary vein. Care should be taken to avoid the LAA, given that inadvertent guide wire or catheter manipulation within this thin‐walled structure can lead to perforation. In the registry compiled by Amin et al, 5 of the 11 complications from PFO closure were from LAA perforation alone.^68^ Once the wire is secured in the pulmonary vein, the catheter is advanced over the wire using a gentle counterclockwise torque. The wire is then exchanged for a stiff 0.035‐inch guide wire to provide support in advancing the sizing balloon and delivery sheath.

### Balloon Sizing

**Figure 9:**
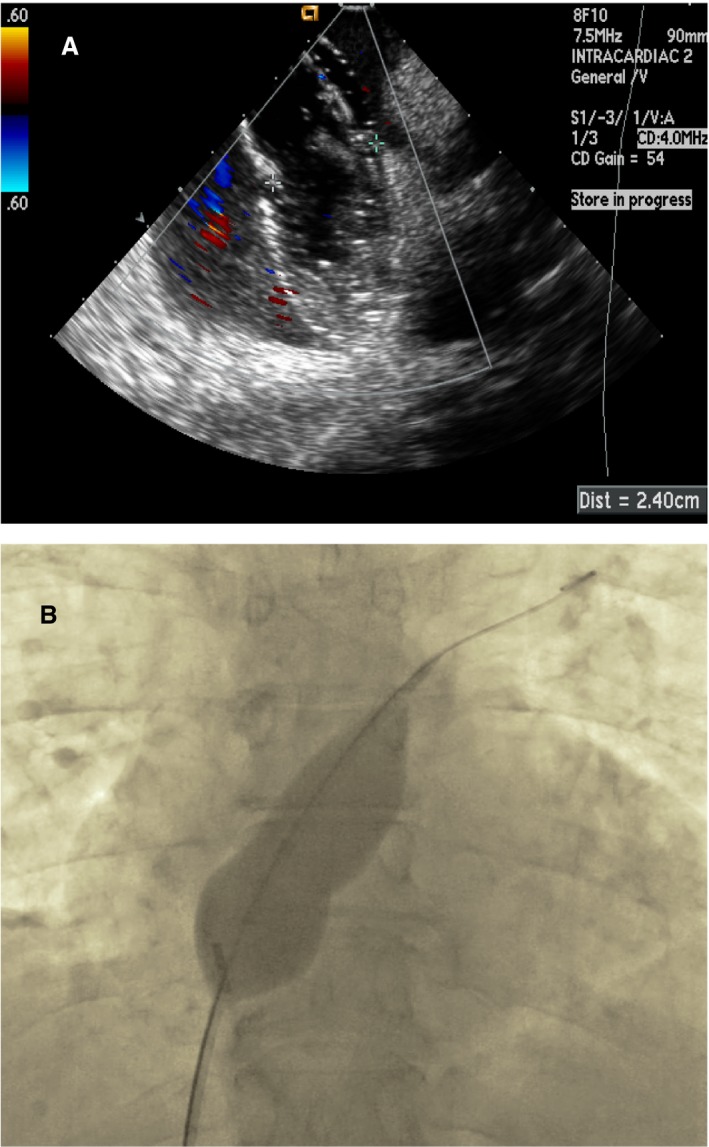
A, Intracardiac image of balloon sizing using stop‐flow technique and (B) fluoroscopic image of an inflated sizing balloon across the patent foramen ovale (PFO).

In most cases, balloon sizing is not necessary for PFO closure. It is often avoided because it can anatomically alter and even enlarge the PFO. Although echocardiographic imaging can usually provide accurate and reproducible data on tunnel length and width, balloon sizing may be helpful to acquire confirmatory assessment of tunnel length or test the compliance of the tunnel before device deployment. The Amplatzer sizing balloon II is commonly used, but any other sizing balloon may suffice. The Amplatzer sizing balloon II comes in 3 sizes. The balloon is then advanced over the stiff guidewire under fluoroscopic and echocardiographic guidance and slowly inflated until the flow across the defect ceases (stop‐flow technique; Figure 9). It is important to avoid inflating the balloon past that point to prevent stretching of the PFO and inaccurate sizing. Measurements should be obtained with the inflated balloon well profiled to avoid foreshortening and inaccurate measurements.

### Device Sizing

For the Amplatzer PFO Occluder, the device size corresponds to the right atrial disc and is available in 3 sizes as described above. Sizing recommendations for the Amplatzer PFO Occluder are presented in Table 4. A minimum distance of 9 mm should be present between the PFO and aortic root or SVC to safely implant the Amplatzer PFO Occluder and minimize risk of erosion or SVC obstruction. The Gore Cardioform Septal Occluder device sizing recommendations include a thorough evaluation with ICE or TEE with color flow Doppler to determine whether there is adequate space to accommodate the chosen device size without impinging vital adjacent structures, such as pulmonary veins, coronary sinus, and mitral and tricuspid valves. Operators should also keep in mind that longer PFO tunnels, larger‐width PFOs, and the presence of ASA often require larger device size to ensure proper closure.

**Table 4:** Amplatzer PFO Occluder Sizing Recommendations

Shortest Distance From Defect to Aortic Root, or Distance From Defect to Superior Vena Cava Orifice	Suggested Amplatzer PFO Occluder Size
≥17.5 mm	35 mm
12.5 to 17.4 mm	25 mm
9 to 12.4 mm	18 mm
<9 mm	Do not implant device

### Device Deployment

**Figure 10:**
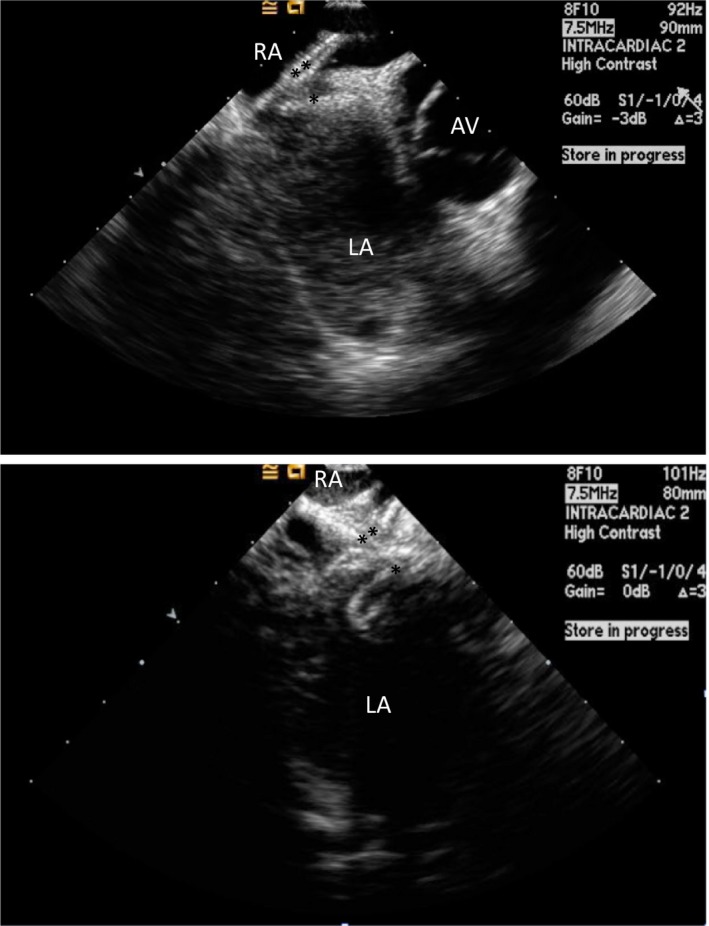
Intracardiac echocardiogram image of the right (**) and left atrial disc (*) of an Amplatzer PFO Occluder (top) and Gore Cardioform Septal Occluder (bottom) before the release of the device. Note the splaying of the discs at the aortic rim. AV indicates aortic valve; LA, left atrium; RA, right atrium.

Although echocardiographic guidance is necessary for almost the entire procedure, its use is most crucial during device deployment and post‐deployment assessment. Before deploying the device, the sheath should be pulled back under fluoroscopic and ICE/TEE guidance to a position in the mid‐left atrium to ensure that the left atrial disc, when deployed, will not become entrapped in the pulmonary vein, LAA, or interact with the left atrial free wall.^13^ To deploy the left atrial disc of the Amplatzer PFO Occluder, the delivery sheath is pulled back slowly over the delivery cable to “unsheath” the device. To deploy the left atrial disc of the Gore Cardioform Septal Occluder, the slider in the handle is pushed forward while withdrawing the delivery catheter. Echocardiographic guidance is critical for this stage of the procedure and should demonstrate that the left atrial disc is free of adjacent structures in the midleft atrium. Once the left atrial disc is deployed, the delivery system is gently pulled as a unit until the left atrial disc engages the atrial septum. Tension on the delivery system is then maintained as the right atrial disc is subsequently deployed. Once the right atrial disc is deployed, the delivery sheath is slightly advanced to relieve tension on the system. This maneuver also flattens the right atrial disc and apposes the device to the atrial septum. Under fluoroscopy, both discs may be visualized in profile using a left anterior oblique with cranial angulation. The typical orientation of the discs at that time is often not parallel because of the tension on the delivery system. The splay and separation between the 2 discs are usually apparent in the superior portion of the septum reflecting the thickness of the septum secundum (Figure 10). If the position is unsatisfactory, the device may be recaptured and redeployed as long as it is attached to the delivery system.

Before releasing the device, several key assessments should be done to ensure successful deployment. A “tug test” is done to assess device stability by gently pulling on the delivery system while watching under fluoroscopy and echocardiography. Any residual shunt should be carefully assessed by color Doppler.

**Figure 11:**
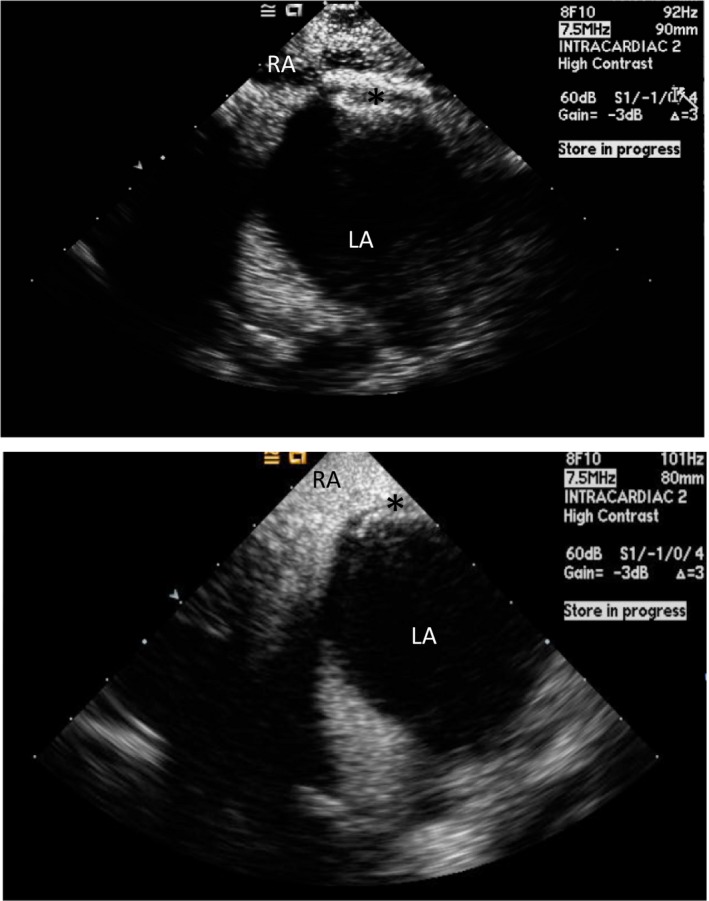
Intracardiac echocardiogram (ICE) image of a released Amplatzer PFO Occluder (top) (*) and Gore Cardioform Septal Occluder (bottom) (*). Agitated saline is seen in the right atrium (RA), but not in the left atrium (LA), confirming the absence of right to left shunt after device deployment.

Once the deployment is deemed satisfactory, the device is released from the delivery system. After the device is released, residual shunting and final device position is once again reassessed using color Doppler and injection of agitated saline (Figure 11). After the device has been successfully placed, the delivery system and introducer sheaths are removed and hemostasis obtained.

### Post‐Procedure Care

Bed rest ranges from 4 to 6 hours depending on sheath size and activated clotting time post‐procedure. Patients are usually kept overnight for observation and monitoring of bleeding complications, atrial arrhythmias, and change in neurological status. An ECG should be obtained post‐procedure to detect any new atrial arrhythmias or evidence of new or worsening atrioventricular blocks.

Optimal type, combination, and duration of antiplatelet therapy post‐PFO closure are unknown. Polzin et al studied the antiplatelet effects of clopidogrel and aspirin after PFO and ASD closure in 140 patients. Their results showed that despite a high incidence of high on‐treatment platelet reactivity (71% to clopidogrel and only 4% to aspirin), no stroke or thrombus formation on the device were detected. This led to the hypothesis that the benefit of additional clopidogrel therapy after PFO closure is questionable and warrants further investigation.^69^ The optimal duration of dual‐antiplatelet therapy post‐PFO closure is also unclear. The most recent clinical trials have variable combinations and duration of therapy. In the RESPECT trial, patients received aspirin 81 to 325 mg plus clopidogrel 75 mg for 1 month, followed by aspirin monotherapy for 5 months.^25^ In the CLOSE trial, patients received aspirin 75 mg plus clopidogrel 75 mg daily for the first 3 months, followed by aspirin or clopidogrel daily for the remainder of the trial.^27^ In the REDUCE trial, patients not previously on clopidogrel received 1 dose of 300 mg before or immediately after the procedure, followed by 75 mg daily for 3 days. After that, antiplatelet therapy was at the discretion of the participating site and consisted of aspirin alone, aspirin plus dipyridamole, or clopidogrel monotherapy.^26^ At our institution, patients not previously on clopidogrel receive 1 dose of 300 mg in addition to aspirin 81 mg on the day of the procedure. This is followed by both aspirin 81 mg daily and clopidogrel 75 mg daily for 6 months. Management of patients who are already taking anticoagulant therapy for other indications, such as deep venous thrombosis or pulmonary embolism, is currently unclear. The contemporary PFO trials did not study patients who are already on chronic anticoagulation. At our institution, patients previously on chronic anticoagulation therapy resume their medication on the same day, and only aspirin 81 mg daily is added for 6 months. The appropriate medical therapy of post‐PFO closure patients requiring chronic anticoagulation for other clinical reasons should be further investigated.

A TTE is done 1 day after the procedure or before discharge. The postprocedure TTE should carefully assess for residual shunts, device instability, evidence of device erosion, deformation of surrounding structures, and evidence of new or worsening pericardial effusion.^13^ Duration and frequency of follow‐up echocardiograms post‐PFO closure is unknown.^13^ In 1 study, in addition to TTE evaluation 1 day after the procedure or before discharge, contrast TTE was repeated at 6 months to document proper device implantation and assess for residual shunts.^70^ Serial echocardiograms thereafter may be done in cases of persistent shunt or larger device implant.^70^

Infection of a PFO device postimplant is exceedingly rare, but several case reports have reported device infection that required either intravenous antibiotics or surgical management.^71^, ^72^ Given the scarcity of data and low infection rate of patients with intracardiac devices (including Amplatzer devices), there is no definitive evidence that postprocedure prophylaxis is warranted in the absence of high‐risk features for intracardiac infection.^73^ Infective endocarditis prophylaxis, if warranted, should be done for the first 6 months only for dental procedures, which involve manipulation of the gingival tissue, perforation of the oral mucosa, or manipulation of the periapical region of the teeth.^73^ Ideally, elective dental procedures should be deferred until 6 months postprocedure, but if not possible, a single dose of amoxicillin 2 mg should be taken 30 to 60 minutes before dental work. As an alternative for patients with a penicillin allergy, clindamycin 600 mg may be given. Prophylaxis is not required for nondental procedures, such as colonoscopy, cystoscopy, and TEE.

## Complications of PFO Closure

Percutaneous PFO closure procedures are generally quite safe when performed by experienced operators. Serious complications are exceedingly rare, and some of these are considered to be attributed to specific design flaws in earlier generation devices. Although transcatheter closure of PFO is recognized as a safe procedure, complications still exist. Anatomical knowledge of the IAS and its surrounding structures, careful manipulation of wires and catheters, constant and clear communication between the operator and echocardiographer, and appropriate use of fluoroscopy and echocardiography for procedural guidance are paramount to mitigate these risks.

### Periprocedural Complications

Although relatively more common than late complications, periprocedural complications are usually benign and reversible. A large study of 307 consecutive patients who underwent PFO closure was done in 2004. The study primarily investigated the periprocedural safety of three PFO devices: the PFO Star (Cardia, Burnsville, MI), Amplatzer PFO Occluder, and CardioSEAL‐Starflex. All procedures used TEE and fluoroscopic guidance. Periprocedural complications occurred in 3% of patients, which included transient ST elevations (n=5), transient ischemic attack (n=2), device dislodgement (n=1), and large residual shunt (n=6). Two patients required surgical removal of the device. One was attributed to significant device misalignment of a PFO Star device, and the other was device‐adherent thrombus in the left atrial surface of a CardioSEAL device.^74^

Vascular injury at the access site is relatively common, albeit mostly benign. It is as frequent as 30% in 1 series, but only 2.4% required surgical intervention.^75^ Other periprocedural reported complications include air embolism, cardiac perforation, and device fracture.

Major complications can occur in 1.2% of cases.^76^ In a separate review of 10 studies, including a total of 1355 patients who underwent transcatheter closure of PFO using different devices, major and minor complications were noted to be 1.5% and 7.9%, respectively.^77^ However, the 10 studies had variable follow‐up and nonstandardized definitions of complications. Table 5 lists the incidences of selected adverse events of the RESPECT trial.

**Table 5:** Selected Adverse Events in the RESPECT Trial

Adverse Event	Device		Medical Therapy		*P* Value
	No. of Events (n=499)	%	No. of Events (n=481)	%	
Atrial fibrillation	6	1.2	4	0.8	0.753
Atrial flutter	1	0.2	0	0	1
Cardiac perforation	1	0.2	0	0	1
Cardiac arrest	1	0.2	3	0.6	0.365
Cardiac thrombus	2	0.4	0	0	0.5
Pericardial tamponade	2	0.4	0	0	1
Pulmonary embolism	12	2.4	3	0.6	0.034
Gastrointestinal bleeding	6	1.2	4	0.8	0.753
Hematoma	1	0.2	0	0	1
Transesophageal echocardiogram related event	1	0.2	0	0	1
Residual shunt requiring closure	2	0.4	0	0	0.5
Deep vein thrombosis	5	1.0	1	0.2	0.218
Myocardial infarction	6	1.2	1	0.2	0.124

Reprinted from Saver et al^25^ with permission. Copyright ©2017, Massachusetts Medical Society. RESPECT indicates Randomized Evaluation of Recurrent Stroke Comparing PFO Closure to Established Current Standard of Care Treatment.

### Device Embolization

Device embolization is a very rare complication following PFO closure and is as low as 0.7%.^63^ Optimal preprocedure planning, intraprocedure imaging, and appropriate device selection are needed to avoid this potentially serious complication. In a case series done by Goel et al, 2 morphological features were associated with device embolization. These features were the presence of a hypermobile septum primum and a thick septum secundum.^63^ No device embolizations occurred in the RESPECT, REDUCE, CLOSE, and DEFENSE‐PFO trials.

### Residual Shunt

Therapeutic success of PFO closure hinges upon complete elimination of the interatrial communication. Thus, historically, the Achilles heel of PFO closure devices has been the persistence of right‐to‐left shunt after deployment. Some of these incomplete closures were observed with the early device designs. Fortunately, newer‐generation devices have significantly reduced this problem. Anzola et al used contrast‐enhanced TCD to detect the presence of right‐to‐left shunt after PFO closure device implant. They detected residual right‐to‐left shunt in 22% of patients after 1 month and 9% of patients at 12 months postprocedure.^78^ Independent predictors of residual shunts after 12 months were the presence of an ASA (odds ratio, 7.6; 95% CI, 1.38–42.35; *P*=0.02), a longitudinal fossa ovalis dimension more than 20.8 mm (odds ratio, 8.5; 95% CI, 1.55–46.95; *P*=0.014),^79^ and the type of device, especially the Helex device (HR, 12.58; 95% CI, 2.57–57.43; *P*=0.002).^80^ Width of the fossa ovalis was not a predictor of residual right‐to‐left shunt. Despite the relatively high incidence of residual shunts postimplant, embolic event rate remained low and did not correlate with recurrent thromboembolic events.^80^ A low incidence of significant residual shunts was observed in the recent major trials, 2 of 499 (0.4%) in the RESPECT trial^23^, ^25^ and 2 of 238 (0.8%) in the CLOSE trial.^27^

### Device Erosion and Cardiac Perforation

Device erosion, and subsequently cardiac perforation, is a life‐threatening complication of transcatheter PFO closure. Amin et al reported an incidence of 0.018% of device erosion from the Amplatzer PFO Occluder from the AGA registry in 2008.^68^ A multicenter survey done by Verma identified 2 patients (0.01%) with perforation out of 13 736 patients who underwent PFO closure. The first occurred immediately after implanting a CardioSEAL device. The second patient had an Amplatzer PFO Occluder 25‐mm device removed for late erosion. Two other patients (0.01%) had Amplatzer devices explanted for pericardial effusion as a result of possible erosion.^81^

Amin identified several key findings in the echocardiogram that increase the risk of erosion and perforation in ASD closures, which can also be applied to PFO closures. These include absence of the aortic rim in multiple views, poor posterior rim consistency, and septal malalignment.^82^

### Nickel Allergy

Nitinol is an alloy of nickel and titanium. It is the primary component of the Amplatzer PFO Occluder, Gore Cardioform Septal Occluder, and most PFO closure devices. Hypersensitivity to nickel has been reported in patients who have PFO closure device implants manifesting as chest pain, shortness of breath, fevers, rash, migraine headaches, or palpitations.^83^ Most of these patients had documented nickel allergy by skin testing and underwent removal of the device with subsequent resolution of symptoms.^84^ Case reports of hypersensitivity to nickel that led to explant of the PFO device were also reported with the PFO Star device and the Helex Septal Occluder.^85^ In the same multicenter survey by Verma, a total of 13 736 device implants were studied. The study focused on the 38 patients who had device removal, of which 14 (37%) had chest pain as the indication for removal. Up to 17% of those patients had a nickel allergy.^81^ It is still unclear whether nickel allergy skin testing on patients with suspected hypersensitivity to nickel before device implant is warranted.

### Pacemaker or Implantable Cardioverter Defibrillator Lead Entrapment

**Figure 12:**
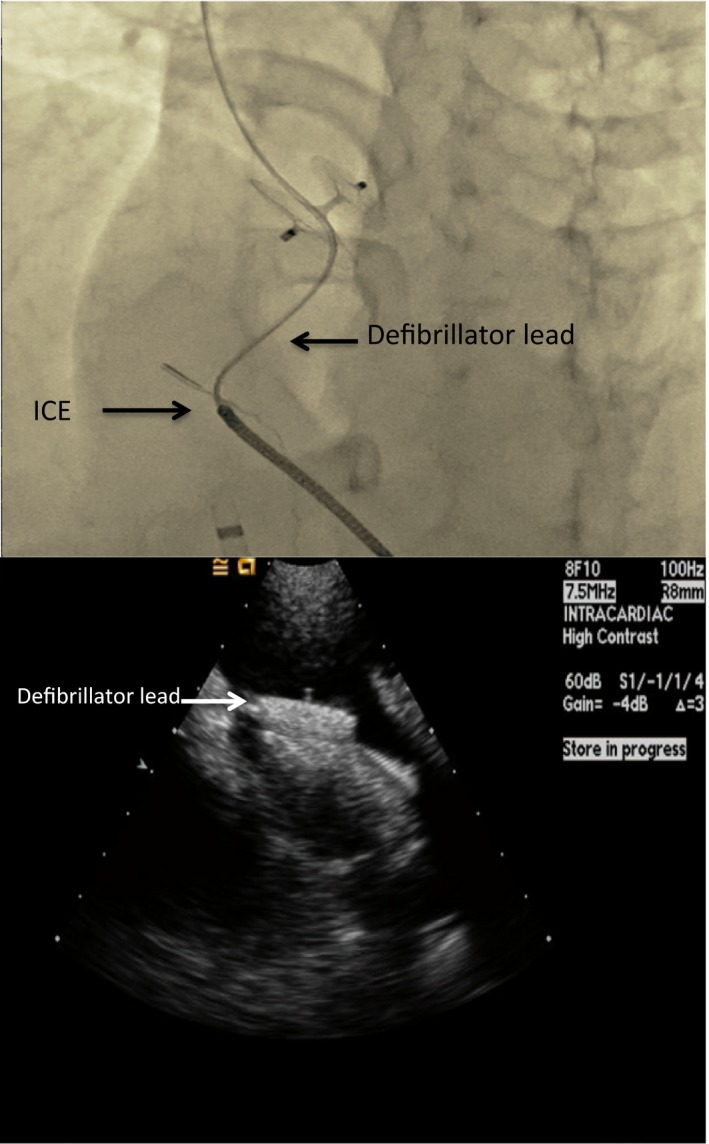
A, Fluoroscopic and (B) ICE images showing a trapped defibrillator lead in between the right atrial disc and the interatrial septum. ICE indicates intracardiac echocardiogram.

As the incidence of pacemaker and implantable cardioverter defibrillator implants increases, it is not unusual for patients being considered for PFO closure to have an existing pacemaker lead. A pacemaker or defibrillator lead in the right atrium can complicate PFO closure procedures through interactions with the delivery system and/or the device. The delivery system may entangle itself with the lead as it passes through the right atrium. Careful removal of the delivery sheath after device deployment should be done under fluoroscopy to avoid further entangling and lead dislodgement. Lead entrapment by the deployed right atrial disc of a PFO closure device is another potential complication that should be recognized before releasing the device (Figure 12). When encountered, the device should be recaptured and repositioned. Encountering this problem after device release is more complicated and requires the use of snare devices and improvised catheter approaches to free the lead from under the right atrial disc, risking dislodging the lead in the process.^86^, ^87^

### Thrombus Formation on the Device

**Figure 13:**
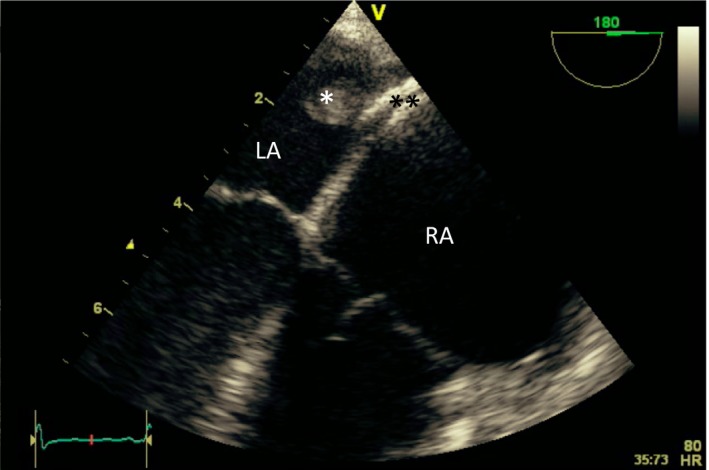
Transesophageal echocardiogram (midesophageal at 180 degrees) showing a thrombus (*) attached to the left atrial disc of an Amplatzer PFO occluder (**). LA indicates left atrium; RA, right atrium.

PFO closure devices, like all foreign body implants, are a potential source of thrombus formation (Figure 13). Fortunately, the incidence of thrombus on PFO closure devices is low. In 1 study, incidence of thrombus formation was 7.1% on the CardioSEAL device; 5.7% on the Starflex device; 6.6% on the PFO Star device; 3.6% on the ASDOS device (Dr Ing, Osypka Corp, Grenzach‐Wyhlen, Germany); 0.8% on the Helex device; and 0% on the Amplatzer device.^88^ In this study, TEE was performed in all subjects at 1 and 6 months. Most of the patients with thrombus (17 of 20) were successfully treated with anticoagulation using heparin or warfarin.^88^ There was no incidence of device thrombus formation in the RESPECT trial patients.^25^ On the other hand, in the REDUCE trial, 2 patients (0.5%) had device‐related thrombosis.^26^

### Atrial Fibrillation

New‐onset periprocedural AF is a known complication of percutaneous PFO closure with an incidence of up to 3.9% from a large series of 1349 patients who underwent PFO closure.^89^ In the RESPECT (Long‐Term Outcomes) trial, new‐onset AF was detected in 1.2% in the closure group and only 0.8% in the medical therapy group (*P*=0.753; Table 5).^25^ The CLOSE trial reported an incidence of 2.5% in patients who underwent PFO closure,^27^ whereas the REDUCE trial reported an incidence of 6.6% for any AF or atrial flutter and 2.3% for serious AF or atrial flutter.^26^ In a large series by Staubach, among patients who developed new‐onset AF post PFO closure, 62.3% were detected within 4 weeks and 15% between 4 weeks to 6 months post‐procedure.^89^ The exact mechanism of new‐onset AF after PFO closure is not known, but several hypotheses have been suggested. Potential mechanisms of AF after PFO closure include intrinsic patient related factors as well as local stretch and irritation from the device itself. The device may lead to a local inflammatory response that can trigger atrial arrhythmias. The device may also cause electrical obstruction causing new reentry circuits in the left or right atrium or both. Diagnosis and treatment of AF after PFO closure should be done promptly. In these patients, there is a higher incidence of left atrial thrombi in patients compared with those without AF. Advanced age as well as use of a Starflex device predicted new‐onset AF.^89^

## PFO Closure Training Requirements

With the recent results from randomized clinical trials, an increase in PFO closure device implants across the United States may be anticipated. To standardize and ensure optimal outcomes, it is recommended that PFO closure be performed in high‐volume and experienced centers. There is a specific cognitive and technical skillset that needs to be mastered before performing PFO closure (Table 6).^90^ After the approval of the Amplatzer PFO Occluder, the FDA clearly mandated a comprehensive physician‐training program for new and experienced operators, including didactic training and case support by experienced proctors for the first cases. The FDA set a minimum of 25 PFO implantation procedures to become certified as an independent operator. Physicians interested in PFO implantation can avail themselves of training opportunities, such as didactics, hands‐on experience, simulators, and viewing live cases being performed by experienced operators. However, formal training in structural heart disease interventions through a dedicated fellowship may be the optimal route in becoming a competent operator for PFO closure device implantation.^51^

**Table 6:** Recommended Knowledge Base and Interventional Skills for Percutaneous Patent Foramen Ovale Closure

Knowledge Base	Interventional Skills
Understanding the natural history of paradoxical thromboembolic events and right‐to‐left shunts through PFO Medical management and guidelines Randomized Clinical Trials data Image interpretation a. Transesophageal echocardiography b. Intracardiac echocardiography c. Cardiac Magnetic Resonance Imaging d. Transcranial Doppler Types of PFO devices and sizes Indications to intervene Contraindications to PFO closure Potential complications	Right and left heart catheterization Crossing a PFO Balloon sizing if appropriate Sheaths, wires, and catheters to use Image guidance a. Transesophageal echocardiography b. Intracardiac echocardiography c. Fluoroscopy Retrieval of embolized devices Acute and long‐term postprocedural care Management of complications

Reprinted from Ruiz et al^90^ with permission. Copyright ©2010, Elsevier. PFO indicates patent foramen ovale.

## Future Directions

Because of the highly variable anatomical morphology of the PFO with respect to size, tunnel length, redundancy of septum, thickness of septum secundum, and relationship to neighboring structures, a single device might not be suitable for optimal treatment of all PFOs. Devices uniquely suited to specific anatomical variants will improve the success rate of the procedure and minimize complications as well as residual shunts.

As the field moves forward, new devices will appear on the landscape that will be uniquely suited for specific anatomical PFO subsets, such as long tunnels or a highly mobile redundant septum. Ultimately, a “no footprint” device may be the most attractive solution, and such devices are currently working their way through the regulatory pathways. Devices uniquely suited to specific anatomical variants will improve the success rate of the procedure and minimize complications and residual shunts.

The presence of a PFO closure device makes future access to the left atrium challenging. With the growth of left‐sided ablation procedures, percutaneous mitral valve interventions and LAA closure, access to the left atrium through the IAS is becoming increasingly important. The presence of a PFO device will make access to the left atrium for such procedures very difficult and has fueled interest in developing a bioresorbable PFO device. In the phase I clinical trial, BEST (BioSTAR Evaluation Study), a novel bioresorbable PFO closure device was deemed feasible, safe, and effective. It was noted that 90% to 95% of the device was absorbed in healthy native tissue.^91^ Although the device is associated with a low complication and embolic event rate, there was a high percentage of residual mild to moderate shunting after 6 months (23.7%).^92^ In 2015, Sievert et al published a study on the effectiveness and safety of the Carag Bioresorbable Septal Occluder. The device is comprised of a poly lactic‐glycolic acid monofilament framework with polyester patches attached. The in vivo study of Carag Bioresorbable Septal Occluder in pigs showed complete endothelialization, which was confirmed histologically. Resorption of the frame material was noted to proceed after implantation.^93^ Further data on these new devices are expected in upcoming human clinical trials.

Along with the FDA approval of 2 devices for PFO closure came a requirement for long‐term follow‐up data through Post Approval Studies. The current clinical program will assess the long‐term safety and effectiveness of the Amplatzer PFO Occluder with 2 nonrandomized studies. The first study will continue to follow active RESPECT trial patients through a minimum of 5 years for the development of new ischemic stroke and other adverse events. The second study is a registry for new patients with cryptogenic stroke undergoing PFO closure with the Amplatzer PFO Occluder device in the United States. These patients will be followed for 5 years. It is anticipated that a similar study will be launched for the Gore Cardioform Septal Occluder.

## Conclusions

Transcatheter PFO closure in the United States has endured a long journey, from the closure of ASDs in dogs in the 1940s to the first 2 FDA‐approved PFO closure devices in the United States on October 28, 2016 (Amplatzer PFO Occluder) and on March 30, 2018 (Gore Cardioform Septal Occluder). Results of the RESPECT extended follow‐up, CLOSE, REDUCE, and DEFENSE‐PFO randomized clinical trials have shown superiority for PFO closure as a nonpharmacological treatment for reducing risk of recurrent ischemic stroke in certain patient subsets when compared with best medical therapy. Governing societies will now be tasked with rewriting guidelines for the management of patients with cryptogenic stroke to reflect the superiority of device closure over medical therapy for this patient population. Furthermore, the term “cryptogenic” as it applies to young patients with cortical ischemic stroke who have no other cause for their stroke other than a PFO should be reclassified as “PFO‐mediated stroke.” As the field moves forward, additional clinical data will provide further refinements on patient selection for this procedure. New devices will appear on the horizon that will provide procedural improvements with even fewer complications and greater success. Physicians must accept and acknowledge the fiduciary responsibility we have toward our patients in ensuring the safe and effective dissemination of this technology to the general public in the spirit of *patient‐centered care*. Proper patient selection, shared decision making, collaboration with our neurology colleagues, and involvement of the patient in all discussions are essential features of a successful PFO closure program and avoid unnecessary procedures. It is important to train future operators in the essential skillsets necessary for the safe and effective application of this technology. Given that most patients treated with this procedure are young and look forward to many years of quality life, it is important to establish mechanisms for long‐term follow‐up of treated patients to ensure adherence to quality metrics and identify underperforming programs. Whether this is best done through a national standardized registry, as is the case for transcatheter aortic valve replacement, percutaneous mitral valve repair, and left atrial appendage occlusion procedures, has yet to be established.

## Disclosures

None.
